# Symptoms of anxiety and depression and their relationship with barriers to physical activity in patients with intermittent claudication

**DOI:** 10.6061/clinics/2021/e1802

**Published:** 2021-01-11

**Authors:** Luciana Ragazzo, Pedro Puech-Leao, Nelson Wolosker, Nelson de Luccia, Glauco Saes, Raphael M. Ritti-Dias, Gabriel Grizzo Cucato, Debora Yumi Ferreira Kamikava, Antonio Eduardo Zerati

**Affiliations:** I Hospital das Clinicas (HCFMUSP), Faculdade de Medicina, Universidade de Sao Paulo, Sao Paulo, SP, BR; II Universidade Nove de Julho, , Sao Paulo, SP, BR

**Keywords:** Intermittent Claudication, Depression, Anxiety, Physical Activity, Barriers, Peripheral Arterial Obstructive Disease

## Abstract

**OBJECTIVES:**

Although the practice of physical exercise in patients with intermittent claudication (IC) is often encouraged, adherence is low. The difficulty in performing physical training may be related to the psychological characteristics of patients with claudication. To verify the association between anxiety and depression symptoms and barriers to physical exercise and walking capacity in patients with IC.

**METHODS:**

One-hundred and thirteen patients with a clinical diagnosis of IC were included in the study. Patients underwent clinical evaluation by a vascular surgeon, answered the Beck Depression Inventory, and Beck Anxiety Inventory tests were applied by the psychologist. The patients performed the 6-minute test and reported their barriers to physical activity practice in a questionnaire.

**RESULTS:**

Patients with signs of depression had a shorter pain-free walking distance (*p*=0.015) and total walking distance (*p*=0.035) compared to patients with no signs of depression. Pain-free walking distance (*p*=0.29) and total walking distance (*p*=0.07) were similar between patients with and without signs of anxiety. Patients with symptoms of moderate to severe depression reported more barriers to physical activity practice compared to patients without signs of depression.

**CONCLUSION:**

Symptoms of anxiety and depression are prevalent among patients with peripheral arterial occlusive disease (PAD). Depression symptoms are associated with personal barriers to exercise, while anxiety symptoms are not. The main barriers to physical activity among patients with IC are exercise-induced pain and the presence of other diseases.

## INTRODUCTION

Depression and anxiety are related to several chronic diseases, including coronary disease (1). Depression is more frequent in the cardiac patient population and is an independent risk factor for worse coronary disease prognosis (2). In addition, the combination of anxiety and depression further increases the risk of cardiovascular events (3). Similar to coronary disease, the major etiology of peripheral arterial occlusive disease (PAD) is atherosclerosis. Thus, the relationship between depression/anxiety and PAD has also aroused interest.

Smolderen et al. (1) studied 628 patients with PAD and found anxiety symptoms in 29%, depression in 30%, and anhedonia in 28% of these individuals. These psychiatric disorders were more prevalent in patients with more severe PAD (1). In addition to controlling the risk factors for atherosclerosis, PAD treatment involves routine physical activity, especially walking (4,5). Arterial obstruction causes pain due to muscle ischemia triggered by exercise, a condition that defines intermittent claudication (IC) and makes walking a painful activity (6,7).

Thus, the initial clinical treatment of PAD depends on an unpleasant practice that is painful to most patients. Barbosa et al. (8) studied 150 patients with IC and observed that the most prevalent barriers to physical activity were personal and environmental. The emotional state might play a role in enhancing these barriers, thereby decreasing the willingness of patients with IC to perform physical activity and consequently aggravate their PAD. However, no study has analyzed the relationship between the barriers to the practice of exercise and the presence of anxiety/depression.

This study evaluated the relationship between anxiety/depression symptoms and the presence of barriers to exercise with regard to the physical activity levels of patients with IC.

## MATERIAL AND METHODS

### Study design and patients

This prospective, cross-sectional study examined 113 patients with IC of atherosclerotic etiology in one or both legs who were treated at the Vascular Surgery Outpatient Clinic between 2016 and 2018. The institutional ethics committee approved this study, and all patients signed an informed consent document prior to inclusion. The inclusion criteria were: decreased intensity or absence of arterial pulse in at least one of the extremities, ankle-brachial index less than 0.90, ≥20% reduction in systolic blood pressure in the ankle immediately after walking the maximum claudication distance in a standardized treadmill test with progressive load (Gardner protocol), and patients without limiting claudication (walking distance >50 m). The following patients were excluded: association of other limiting factors such as osteoarticular disease in the lower limbs, major lower limb amputation, severe chronic obstructive pulmonary disease, decompensated coronary disease, previous diagnosis of depression or anxiety, use of antidepressants and/or anxiolytics.

### Intermittent claudication assessment

At the initial consultation, the participants’ medical histories were taken, and a general and vascular physical examination was performed to obtain anthropometric data, including a measurement of the ankle brachial index. The patients also reported their physical activity routines. After this consultation, the patients performed the 6-minute walk test (6MWT) to measure their pain-free walking distance and maximum walking distance. Participants also filled a questionnaire to assess the barriers to physical activity, and the Beck Depression Inventory (BDI) to assess their depression and anxiety symptoms. Based on these symptoms, the patients were classified into two groups: asymptomatic or symptomatic.

The ankle brachial index was calculated by dividing the ankle systolic blood pressure by the brachial systolic blood pressure, which permits an assessment of the level of hemodynamic changes of the lower limbs (9). This measure was taken in one or both legs with PAD (10). Systolic ankle and brachial pressures were obtained using a mercury sphygmomanometer and a doppler ultrasound (Martec DV 6000, Ribeirão Preto, Brazil). In the 6MWT, patients walked down a flat 30-meter hallway for 6 minutes. Patients were encouraged to complete as many laps as possible, even if the patient needed to rest. It was important that the patient maintained his or her typical walking rhythm. The distance to the initial pain and the total distance over 6 minutes were recorded (11).

The barriers analyzed refer to the difficulties encountered by patients that prevent them from practicing physical activity. To evaluate the perceived difficulty, an adapted version of the Neighborhood Environmental Walkability Scale (NEWS) (12) was used. This was previously validated by Malavasi et al. (13) and later used in IC patients (7).

### Barriers to physical activity assessment

Personal barriers to physical activity were assessed using questions that assessed the following items: fatigue, lack of time, no one to accompany patients while walking, lack of money to practice physical activity, lack of knowledge, and uncertainty regarding the benefits of exercise, lack of energy, pain induced by walking, and the need to rest due to pain. This questionnaire was validated for use among the elderly and was administered by the same physician (8). Patients answered yes or no to all questions regarding barriers that might affect their ability to engage in physical activity.

### Depression/anxiety questionnaires

For a better understanding, symptoms of depression and anxiety were investigated with an interview with a psychologist who applied two tests to assess the symptoms of depression and anxiety. The BDI and the Beck Anxiety Inventory (BAI) were chosen for this analysis because of their ease of application, validation in other diseases, and other questionnaires (14). The BDI is a self-reported questionnaire consisting of 21 items scored on a 4-point scale ranging from 0 to 3, which measures the severity of depressive symptoms. The total score ranges from 0 to 63, with 0 to 13 indicating minimal depression, 14 to 19 indicating mild depression, 20 to 28 indicating moderate depression, and 29 to 63 indicating severe depression.

Anxiety symptoms were investigated using the BAI. The BAI is a list that describes 21 anxiety symptoms. Patients assessed the symptoms that they experienced during the past week and reported how much these symptoms bothered them. The items were scored on a 3-point scale ranging from 0 to 3, and the total score ranges from 0 to 63, where 0-9 indicate normal or no anxiety, 10-18 indicate mild-to-moderate anxiety, 19-29 indicate moderate-to-severe anxiety, and 30-63 indicates severe anxiety.

### Statistics

SPSS version 17.0 (Chicago, USA) was used to analyze the data. The results are presented as means, standard deviations, and frequencies. Data normality was confirmed using the Shapiro-Wilk test and visual inspection of the histograms. Categorical variables were tested using the Pearson's chi-square test, and Student's t-test was used for independent samples. A *p*-value of ≤0.05 was adopted as the significance level.

## RESULTS

The characteristics of the sample, which was predominantly male (67.8%) with a mean age of 66.7 years, depressive symptoms were more frequent than anxious symptoms, as detailed in Table 1.

**Figure 1:**
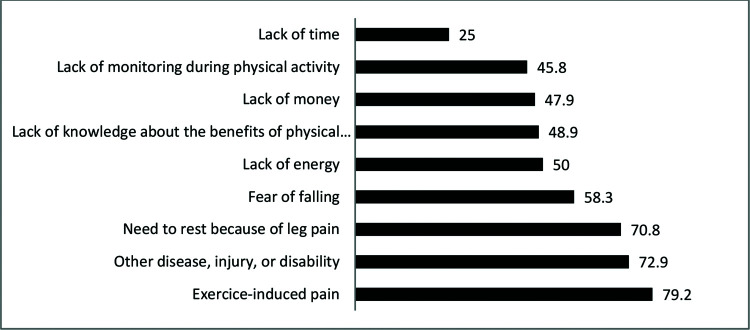
Prevalence of personal barriers to physical activity among patients with IC.

Fig. 1 shows the prevalence of personal barriers to walking. The two most prevalent barriers were pain induced by exercise and the presence of other diseases, injuries, or disabilities that impeded physical activity.

Patients with symptoms of depression had a shorter pain-free walking distance (*p*=0.015) and total walking distance (*p*=0.035) than those without symptoms of depression. The pain-free walking distance (*p*=0.29) and the total walking distance (*p*=0.07) of patients with and without symptoms of anxiety were similar (Table 2).

**Figure 2:**
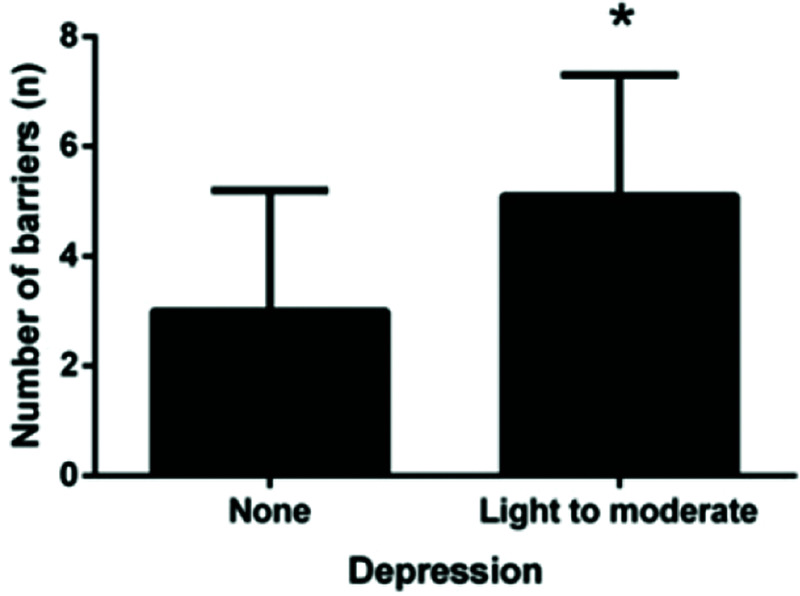
Comparison of the number of barriers according to the depression symptoms; *p<0.005.

**Figure 3:**
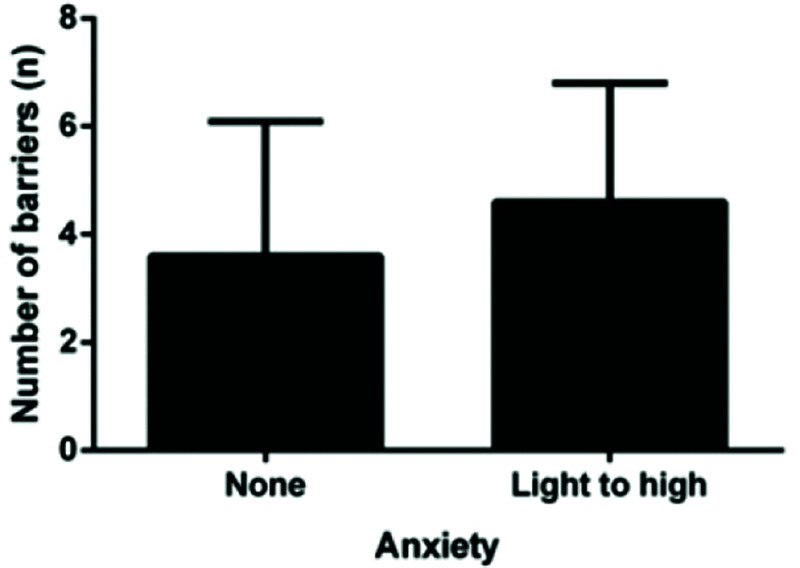
Comparison of the number of barriers according to the anxiety symptoms.

A comparison of the number of barriers between the presence of anxiety/depression symptoms is shown in Figs. 2 and 3. We observed that patients with symptoms of depression had significantly more barriers (*p*<0.005). This pattern was not observed among patients with symptoms of anxiety. Most patients reported more than one barrier, so the data overlap.

Table 3 shows the relationship between barriers to physical activity and depressive symptoms. Only three barriers were more significant among patients with depression than among those without depression.

Table 4 presents the association between barriers to physical activity and anxiety symptoms. There was no difference in the barriers present between patients with and without anxiety.

## DISCUSSION

IC is the clinical manifestation of PAD that typically hinders patients’ usual activities by decreasing their ability to walk daily (15,16) and consequently reduce their quality of life (17). The initial treatment of IC is often clinical because, with conservative treatment, most patients experience improvement or stability in their clinical manifestations combined with a low risk of amputation. This treatment consists of the use of antiplatelet therapy and statins, the modification of aggressive risk factors (*i.e*., smoking, hyperlipidemia, and hypertension), and physical activity (18). When clinical treatment does not produce results, conventional or endovascular revascularization becomes an alternative (18).

The sex ratio of our sample (67.9% male, 31.1% female) was comparable to that of other studies that have evaluated patients with IC (19). The high rates of hypertension, smoking, diabetes, and acute myocardial infarction in our sample were similar to those expected for patients with PAD (20).

Psychological disorders such as depression and anxiety are significantly correlated with smoking. Several hypotheses have been proposed to explain the high rates of smoking among people with depression and anxiety (21). These hypotheses include the following: the individual smokes to alleviate their symptoms and therefore, the symptoms of depression and anxiety stimulate smoking; alternatively, smoking might lead to depression or anxiety through their effects on an individual's neurocircuits, which increases susceptibility to environmental stressors (21). In our sample, the incidence of active smoking was 19.5%, and this behavior was more frequent among patients with depression; however, no significant difference was observed with regard to patients without depression. The same result was observed with regard to patients with anxiety. It was not possible to establish a causal relationship between psychological symptoms and active smoking.

The daily physical activity of patients with IC decreases as the severity of PAD increases due to functional status deterioration (17). As a result of this process, patients tend to avoid physical activities because they lead to pain. Once patients stop adhering to regular exercise, they enter a vicious circle that can lead to a progressive worsening of functional status and quality of life (22).

Although walking is one of the most accessible forms of physical activity and can easily be incorporated into patients' routines, especially those with IC, these patients do not easily adhere to treatment (23). Understanding the barriers associated with this low tendency to adhere to treatment can help solve a major problem with treatment.

The barriers analyzed here refer to the difficulties encountered by patients which prevent them from practicing physical activity. These barriers were studied through a previously validated questionnaire (8). The analysis of the barriers to the practice of physical activity in our patients, regardless of mood, revealed that the most prevalent barrier was related to claudication symptoms (difficulty in walking with pain), which is consistent with previous studies (8,24,25). Patients with PAD are prone to lack of exercising and a sedentary lifestyle, which is the reverse of the therapeutic proposal. Other associated diseases such as arthrosis and cataracts as well as pulmonary and cardiac symptoms, are important barriers that hamper an exercise program. Several studies have demonstrated an association between anxiety/depression and cardiovascular disease (17,19,21). The structural brain abnormalities resulting from PAD might predispose patients to geriatric depressive symptoms known as “vascular depression” (26). In addition, the number of studies that have assessed anxiety and depression symptoms in people with PAD has been increasing.

Several tests can be applied to identify patients’ moods (14). The tests are mostly self-assessments in which the patient reads multiple-choice questions and selects the best answer. The BDI was chosen by our group as it is among the most used and validated scales for various purposes such as the analysis of depressive symptoms after stroke, and it is equivalent to other tests (14,27). Definitive cut-offs to classify anxiety or depression as “minimal”, “mild”, “moderate”, and “severe” are not defined in the literature. In our study, we decided to use lower cutoff values to increase the sensitivity to detect anxiety and depressive symptoms.

Studies have shown a depressive symptom prevalence of approximately 16% among patients with IC (28). We observed a higher prevalence in our sample (40%). This difference is most likely due to the exclusion of patients previously treated with antidepressants. When patients are treated, they might not report mood swings.

Anxiety has recently received increased attention, especially with regard to heart patients because both anxiety and depression have a strong relationship with the increased risk of adverse outcomes during surgery (3). Anxiety is more prevalent among patients with severe PAD (critical ischemia). Smolderen et al. (1) demonstrated that anxiety was less prevalent in asymptomatic patients and increased progressively with worsening symptoms. Thus, patients with pain at rest had a higher degree of anxiety than those who were asymptomatic. Therefore, we expected that anxious patients would have shorter (pain-free and total) walking distances, and the reporting of pain symptoms would occur in advance because anxiety about having pain would reduce the pain-free distance, but this result was not evident in our series. Patients with and without anxiety had similar walking limitations, which is consistent with what we expected, the total distance would not change because it is more related to physical disability resulting from arterial obstruction.

Patients with depression walk less than non-depressed patients, most likely because they exercise less. This finding was noted when we observed that patients with depression reported more barriers than non-depressed patients. Non-depressed patients are likely able to overcome these barriers and exercise regularly, thereby generating more expressive results with regard to walking distances. Non-depressed patients walk more than depressed patients, unlike anxious patients. Anxious patients do not report more barriers than non-anxious patients; therefore, they likely exercise regularly, regardless of their mood.

Interestingly, the lack of money, fear of falling, and worsening of PAD were the most important barriers among patients with depression. The walking instructions provided by the doctors included walking around the house, on flat streets, or in parks without slopes or hills, and avoiding rough terrain and bumpy sidewalks (which is not always feasible). An earlier study found that individuals who did not live near parks were 35% more likely to be inactive (8). Patients who need to leave their environment and go to other places to exercise, which is associated with a lack of money, might have personal reasons to engage in physical activity.

Depressive symptoms alone can inhibit healthy behaviors and lead to functional declines through psychological and/or biological pathways. In general, the depressive symptoms of patients with IC can generate greater daily limitations than claudication alone.

Considering the high prevalence of depressive symptoms and its relationship to the barriers that hamper clinical treatment (*i.e*., daily physical activity), the treatment of depression should most likely result in improved walking distances and quality of life.

Our study had certain limitations, including the questionnaires used to investigate the barriers to physical activity. These questionnaires were adapted for patients with PAD; however, only minor changes were made (8), and these changes might not have altered the results regarding both the prevalence of barriers and their association with physical activity level. Another limitation was the number of patients recruited, which was small primarily because most eligible patients received treatment from other medical specialties or were taking psychotropic medications.

## CONCLUSIONS

Symptoms of anxiety and depression are prevalent among patients with PAD. Depression symptoms are associated with personal barriers to exercise, while anxiety symptoms are not.

The main barriers to physical activity among patients with IC are exercise-induced pain and the presence of other diseases.

## AUTHOR CONTRIBUTIONS

Ragazzo L contributed in article’s conception, researcher, manuscript development and accountability. Puech-Leao P and de Luccia N contributed in critical revision and approval of the manuscript. Wolosker N contributed in article’s conception and critical review. Saes G contributed in researcher and critical revision. Ritti-Dias RM and Kamikava DY contributed in researcher and statistical analysis. Kanikava D participated as researcher. Zerati AE contributed in article’s conception, critical review and approval of the manuscript.

## Figures and Tables

**Table 1:** The characteristics of the sample (n=113).

Variables	Values
Mean±standard deviation	
Age, y	66.7±9.4
Body mass index, kg/m^2^	27.6±5.6
Ankle brachial index	0.54±0.18
Claudication onset time, s	145±87
Peak walking, m	346±90
Frequency	
Sex, % male	67.8
Current smoking, % yes	16.7
Diabetes mellitus, % yes	52.4
Hypertension, % yes	86.9
Dyslipidemia, % yes	84.5
Coronary artery disease, % yes	36.9
Depression	
None	60.0
Mild	22.5
Moderate	17.5
Severe	-
Anxiety	
None	73.8
Mild	15.0
Moderate	7.5
Severe	3.7

**Table 2:** Walking capacity according to the presence of depressive and anxiety signs.

	Symptoms of depression			Symptoms of anxiety		
	No	Yes	*p*	No	Yes	*p*
Pain free walking distance, m	159±87	119±63	0.015	148±84	129±71	0.29
Total walking distance, m	347±71	313±71	0.035	343±84	309±85	0.07

**Table 3:** Association between barriers to physical activity and depressive symptoms (n=80).

	Depression indicators		
Variables	None (n=48)	Light to moderate(n=32)	*p*
Lack of time, %	22.9	31.2	0.41
Lack of energy, %	37.5	71.9	<0.01
No one to accompany patients during physical activity, %	25.0	50.0	0.02
Lack of money, %	20.8	59.4	<0.01
Have other disease or disability, %	47.9	62.5	0.20
Lack of knowledge and uncertainty regarding the benefits of physical activity, %	16.8	38.7	0.03
Exercise-induced pain, %	56.2	75.0	0.09
Need to rest because of leg pain, %	45.8	56.2	0.36
Fear of falling or worsening the disease, %	31.2	62.5	<0.01

**Table 4:** Association between barriers to physical activity and anxiety symptoms (n=80).

	Anxiety indicators		
Variables	None (n=59)	Mild to Severe (n=21)	*p*
Lack of time, %	22.0	38.1	0.15
Lack of energy, %	49.2	57.1	0.53
No one to accompany patients during physical activity, %	28.8	52.4	0.05
Lack of money, %	32.2	47.6	0.21
Have other disease or disability, %	52.5	57.1	0.72
Lack of knowledge or uncertainty regarding the benefits of physical activity, %	25.4	25.0	0.97
Exercise-induced pain, %	62.7	66.7	0.75
Need to rest because of leg pain, %	47.5	57.1	0.45
Fear of falling or worsening the disease, %	40.7	52.4	0.35
